# Immune recovery following switch from EFV-based regimens to bictegravir/emtricitabine/tenofovir alafenamide in virologically suppressed immunological non-responders: a 144-week real-world cohort study in China

**DOI:** 10.3389/fimmu.2026.1862837

**Published:** 2026-06-09

**Authors:** Honghong Yang, Qing Yu, Mei Li, Min Liu

**Affiliations:** Division of Infectious Diseases, Chongqing Public Health Medical Center, Chongqing, China

**Keywords:** antiretroviral therapy (ART), bictegravir/emtricitabine/tenofovir alafenamide (BIC/FTC/TAF), efavirenz (EFV), immune reconstitution, immunological non-responders (INR)

## Abstract

**Objective:**

To investigate the prevalence of immunological non-responders (INRs) among people living with HIV (PLWH) administered long-term antiretroviral therapy (ART) in Chongqing, China, and to evaluate whether switching from an efavirenz (EFV)-based regimen to bictegravir/emtricitabine/tenofovir alafenamide (BIC/FTC/TAF) was associated with immune recovery in INR patients.

**Methods:**

This retrospective real-world cohort study was conducted at Chongqing Public Health Medical Center. PLWH administered continuous two-nucleoside reverse transcriptase inhibitors (NRTIs) plus EFV therapy for ≥4 years, who achieved sustained viral suppression (HIV RNA <50 copies/mL) but maintained CD4 cell counts <350 cells/μL were defined as INRs according to Chinese guidelines. PLWH were grouped according to whether they continued EFV-based therapy (EFV group) or switched to BIC/FTC/TAF between January and December 2022. Changes in CD4 cell counts, CD4/CD8 ratios, immune reconstitution rates, and virological suppression were assessed at weeks 48, 96, and 144.

**Results:**

Among 4,613 PLWH with long-term treatment, 1,379 were identified as INRs, yielding a prevalence of 29.9%. A total of 446 INRs consistently on a two-NRTI plus EFV regimen (no prior ART switches) were included (median age 50.0 years; median ART duration 69.5 months), of whom 131 switched to BIC/FTC/TAF and 315 continued EFV-based therapy. Compared with the EFV group, the BIC/FTC/TAF group showed greater CD4 cell increases from switching baseline at weeks 48 (66.0 vs. 29.0 cells/μL, P<0.001), 96 (82.5 vs. 46.0 cells/μL, P=0.001), and 144 (82.5 vs. 43.5 cells/μL, P<0.001). Immune reconstitution rates were higher in the BIC/FTC/TAF group at week 48 (34.4% vs. 24.1%, P=0.027) and week 144 (43.8% vs. 32.8%, P=0.005). The immunological benefit was primarily observed in patients aged ≤50 years. Virological suppression rates remained high in both groups.

**Conclusion:**

INRs remain prevalent among PLWH with long-term treatment in southwestern China. In this observational cohort, switching from long-term EFV-based regimens to BIC/FTC/TAF was associated with greater CD4 cell recovery in virologically suppressed INRs, particularly among patients aged ≤50 years. These findings should be interpreted as exploratory and require confirmation in prospective studies.

## 1 Introduction

Antiretroviral therapy (ART) effectively suppresses viral replication, increases CD4 cell count, and promotes immune reconstitution. However, approximately 10–40% of individuals receiving effective ART fail to achieve adequate immunological recovery (1), and commonly referred to as immunological non-responders (INRs). Due to their incomplete immune recovery, INRs face a significantly elevated risk of morbidity and mortality across a range of conditions (e.g., metabolic, cardiovascular, hepatic, renal diseases, non-AIDS malignancies, and neurocognitive disorders) compared to immunological responders (IRs), which severely threaten the lives of people living with HIV (2–5).

Currently, therapeutic approaches to restore immune response in INRs remain undefined. Several immunomodulatory or adjunctive strategies, including IL-7 (6), metformin (7), hydroxychloroquine (8), chloroquine (9), and other investigational interventions such as (5R)-5-hydroxytriptolide (10, 11), have been explored in selected INR populations, but none has been established as routine clinical practice. Similarly, although some observational studies have suggested that ART regimen composition may be associated with differences in immune recovery (12–14), such evidence is limited by non-randomized designs and potential confounding. Randomized clinical trial data have not consistently demonstrated clinically meaningful superiority of specific ART regimens in improving CD4 cell recovery or CD4/CD8 ratio among virologically suppressed individuals. Accordingly, current international guidelines do not recommend routine ART switching solely to improve immune recovery in INRs (15). Therefore, whether ART optimization may be associated with improved immune recovery in selected INR populations remains uncertain.

In China, more than 80% of PLWH in China receive free tenofovir/zidovudine + lamivudine + efavirenz under the National Free Antiretroviral Treatment Program (NFATP) (16). Since the year 2021, the cost of integrase strand transfer inhibitors (INSTIs). including bictegravir/emtricitabine/tenofovir alafenamide (BIC/FTC/TAF), has progressively decreased and is currently covered by Chinese medical insurance, providing a substantial reimbursement policy; consequently, an increasing proportion of people living with HIV (PLWH) currently select INSTI-based regimens. In China, the prevalence rates of late diagnosis and delayed treatment are high (17, 18), and the incidence of INRs may consequently be high. Chongqing, an essential regional hub of south-western China, is more heavily affected by the HIV/AIDS epidemic compared with other regions in China. However, the success of immune reconstitution in Chongqing remains unknown. In addition, some real-world studies have reported immune parameter changes after switching to second-generation INSTI-based regimens in virologically suppressed PLWH (19, 20). However, whether similar changes occur among INRs, particularly those receiving long-term EFV-based regimens, remains unclear. Therefore, this study aimed to investigate the prevalence of INRs in Chongqing and to evaluate whether switching from long-term EFV-based regimens to BIC/FTC/TAF was associated with differences in immunological recovery among virologically suppressed INRs.

## 2 Methods

### 2.1 Study design

This was an observational retrospective, real-world cohort study conducted at Chongqing Public Health Medical Center (CPHMC) in China. PLWH who had received continuous two-NRTIs plus EFV therapy for ≥4 years, achieved sustained viral suppression (HIV RNA <50 copies/mL), but maintained CD4 cell counts <350 cells/μL were defined as INRs according to the latest Chinese guidelines for diagnosis and treatment of acquired immunodeficiency syndrome (15). Patients were then divided into two groups according to whether their ART regimen was switched from January to December 2022: the Continued original regimen (EFV-group) and switched to BIC/FTC/TAF groups. Because treatment switching was based on physician–patient decision-making in routine clinical practice rather than random assignment, the study findings should be interpreted as observational associations.

### 2.2 Data collection

Baseline demographic and clinical characteristics, including age, sex, time before ART, CD4 cell count, HIV-RNA viral load (VL), hepatitis B virus (HBV), hepatitis C virus (HCV) infection, syphilis and opportunistic infections, were extracted from electronic medical records and laboratory databases. Data were collected at switching baseline (i.e., the time point when patients switched antiretroviral regimens) and follow-up visits, which occurred at 48 weeks, 96 weeks, and 144 weeks (with a 4-week window). The variables included HIV-RNA VL, CD4 cell count, CD4/CD8.

### 2.3 Outcomes of interest

The primary outcomes included changes from switching baseline in CD4 cell count and CD4/CD8 ratio at weeks 48, 96, and 144, as well as the proportion of patients achieving immune reconstitution success, defined as a CD4 cell count ≥350 cells/μL according to Chinese guidelines (20). Longitudinal group-by-time interaction analyses were additionally performed to evaluate whether immune recovery trajectories differed between groups over the follow-up period. The secondary outcome was virological suppression during follow-up.

### 2.4 Statistical analysis

Statistical analysis was performed with the SPSS software (version 23.0; IBM Corp., Chicago, IL, USA). Categorical data were expressed as frequency (percentage) and compared via the χ2 test or the Fisher’s exact test. Normally distributed data were expressed as mean ± standard deviation and compared via the independent samples t-test. Data with skewed distribution were expressed as median (P25, P75) and compared by non-parametric tests. A P-value <0.05 was considered to indicate statistical significance. Longitudinal interaction analyses were performed to assess group-by-time interactions for CD4 cell count and CD4/CD8 ratio recovery during follow-up.

## 3 Results

### 3.1 Study population

**Figure 1:**
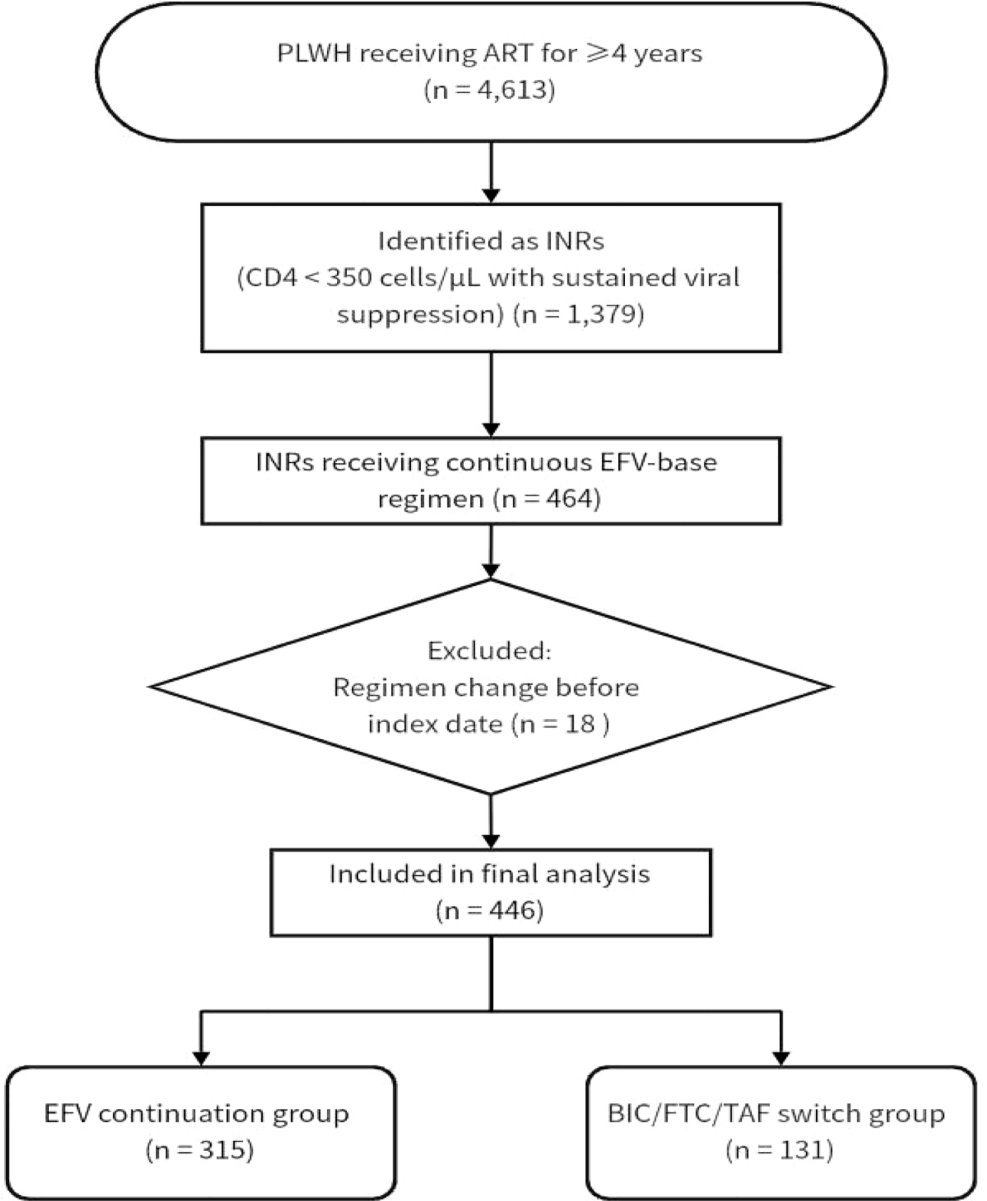
Study flowchart.

From January to December 2022, a total of 4,613 patients had been receiving antiretroviral therapy (ART) at CPHMC for over 4 years. Among them, 1379 PLWH were identified as INRs, yielding an INRs rate of 29.9% (1379/4613). Of the 1,638 patients who had consistently been on a two-NRTI plus EFV regimen (no prior ART switches), 464 were INRs, accounting for 28.3% of this subgroup. After excluding 18 patients (who switched to other ART regimens), 446 were finally included in the analysis. Of these, 315 continued the two-NRTI plus EFV regimen, while 131 were switched to BIC/FTC/TAF. The patient enrollment process is illustrated in **Figure 1**.

The clinicodemographic features of all participants are summarized in **Table 1**. The majority of the 446 patients were male (378/446, 84.8%). The median age of patients was 50.0 years (interquartile range (IQR) 38.0-61.0), with 51.1% under the age of 50 years. Participants received a median of 69.5 months of ART, ranging from 56.7 to 90.8 months. The median CD4 cell count at switching baseline was 264.5 (212.3–303.00) cells/ul. The median switching baseline CD4/CD8 ratio was 0.46 (0.34–0.64), comparable between the two groups. Totally 56 patients (12.6%) had syphilis, 44 (9.9%) had hepatitis B, and 6 (1.3%) had hepatitis C. The baseline clinicodemographic characteristics exhibited a general homogeneity across both groups.

**Table 1:** Baseline characteristics of the assessed PLWH.

Characteristic	Total (N = 446)	EFV-group (n = 315)	BIC/FTC/TAF-group (n = 131)	P
Sex, n (%)				0.084
Male	378 (84.8%)	261 (82.9%)	117 (89.3%)	
Female	68 (15.2%)	54 (17.1%)	14 (10.7%)	
Age, n (%)				0.060
≤50 years	228 (51.1%)	152 (48.3%)	76 (58.0%)	
>50 years	218 (48.9%)	163 (51.7%)	55 (42.0%)	
Opportunistic infections, n (%)	54 (12.1%)	43 (13.7%)	11 (8.4%)	0.121
Hepatitis B co-infection, n (%)	44 (9.9%)	36 (11.4%)	8 (6.1%)	0.086
Hepatitis C co-infection, n (%)	6 (1.3%)	5 (1.6%)	1 (0.8%)	0.676
Syphilis co-infection, n (%)	56 (12.6%)	45 (14.3%)	11 (8.4%)	0.087
ART duration (months)	69.5 (56.6, 90.8)	68.4 (56.3, 89.9)	72.4 (56.9, 93.5)	0.449
HIV-1 RNA at baseline (log10copies/mL)	4.9 (4.1, 5.5)	4.9 (4.3, 5.5)	4.7 (3.6, 5.4)	0.055
CD4 cell counts at baseline (cells/μL)	80.5 (30.3, 149.0)	78.0 (30.5, 140.0)	85.0(30.0, 163.0)	0.437
CD4 cell counts at switch baseline	264.5 (212.3, 303.0)	268.0 (219.0, 306.0)	248.0 (207.0, 298.0)	0.101
CD4/CD8 at switch baseline	0.46 (0.34, 0.64)	0.48 (0.35, 0.65)	0.45 (0.32, 0.60)	0.095

Switching baseline represents the time point when patients switched antiretroviral regimens.

### 3.2 Comparison of immunological responses

#### 3.2.1 CD4 cell count recovery

**Figure 2:**
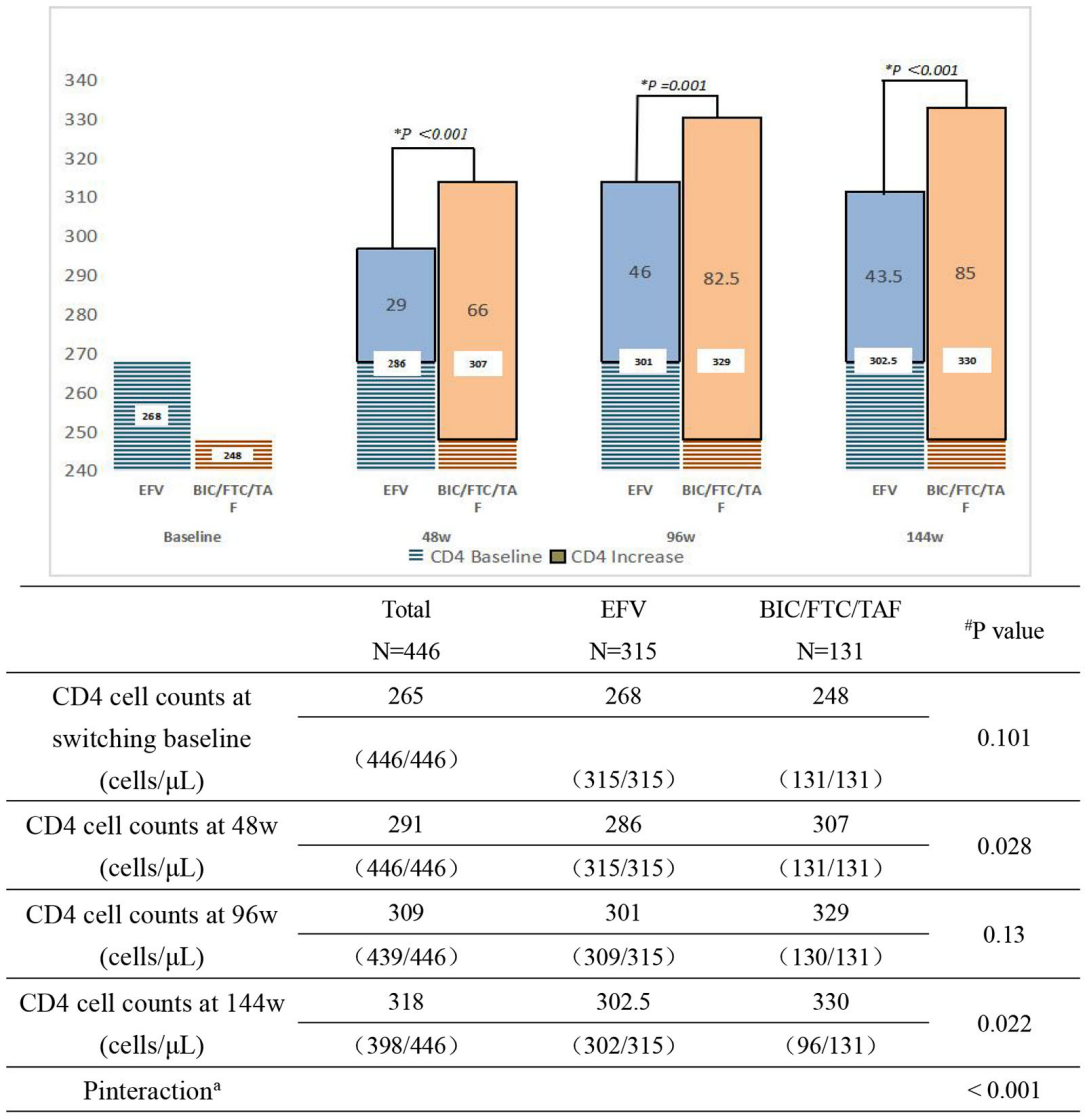
Comparison of cumulative changes in CD4 counts. *P values indicate comparisons of changes from switch baseline between the two groups. ^#^P values indicate comparisons of absolute values between the two groups at the corresponding time point. ^a^Pinteraction values were derived from longitudinal group-by-time interaction analyses evaluating differences in recovery trajectories between groups over time.

CD4 cell counts were assessed at switching baseline and at weeks 48, 96, and 144. At week 48, the median CD4 cell count was higher in the BIC/FTC/TAF group than in the EFV group (307.0 [248.5–363.5] vs. 286.0 [232.5–348.0]; P = 0.028), with a greater median increase from switching baseline (66.0 [18.0–119.5] vs. 29.0 [-14.0–80.0] cells/μL; P < 0.001). Although absolute CD4 cell counts at week 96 were not significantly different between groups (329.0 [235.5–409.8] vs. 301.0 [241.0–383.0]; P = 0.130), greater increases from switching baseline continued to be observed in the BIC/FTC/TAF group (82.5 [25.5–135.8] vs. 46.0 [-3.0–104.0] cells/μL; P = 0.001). At week 144, the BIC/FTC/TAF group showed higher median CD4 cell counts (330.0 [260.5–415.5] vs. 302.5 [240.0–376.0]; P = 0.022) and greater cumulative increases from switching baseline (82.5 [25.5–135.8] vs. 43.5 [-4.8–102.8] cells/μL; P < 0.001). Longitudinal analysis demonstrated a significant group-by-time interaction effect for CD4 cell recovery (Pinteraction < 0.001), suggesting different CD4 recovery trajectories between groups over the 144-week follow-up period (**Figure 2**).

#### 3.2.2 CD4/CD8 ratio recovery

**Figure 3:**
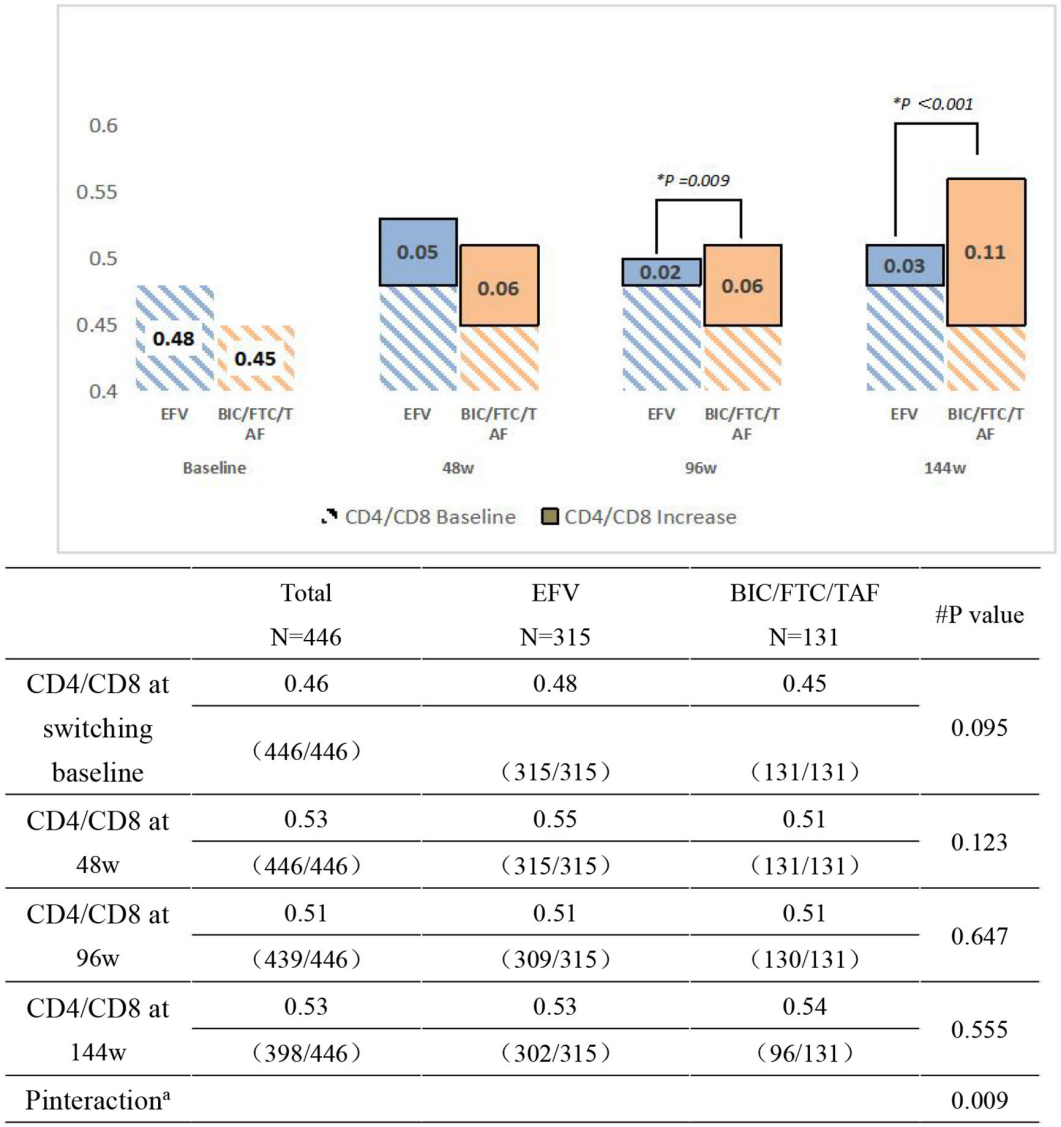
Comparison of cumulative changes in CD4/CD8 ratio. *P values indicate comparisons of changes from switch baseline between the two groups. ^#^P values indicate comparisons of absolute values between the two groups at the corresponding time point. ^a^Pinteraction values were derived from longitudinal group-by-time interaction analyses evaluating differences in recovery trajectories between groups over time.

No statistically significant differences in absolute CD4/CD8 ratios were observed between groups at switching baseline or during follow-up. Similarly, no significant difference in changes from switching baseline was observed at week 48. However, greater increases from switching baseline were observed in the BIC/FTC/TAF group at weeks 96 and 144. Longitudinal analysis demonstrated a significant group-by-time interaction effect for CD4/CD8 ratio recovery, suggesting differing longitudinal recovery patterns between groups despite the absence of significant between-group differences in absolute CD4/CD8 ratios (**Figure 3**).

#### 3.2.3 Rates of immunologic recovery success

All patients were INRs at switching baseline. At week 48, a significantly higher proportion of patients in the BIC/FTC/TAF group achieved immune reconstitution success (CD4 ≥350 cells/μL) compared with the EFV group (34.4% vs. 24.1%; P = 0.027). This numerical advantage for the BIC/FTC/TAF group persisted at subsequent time points, with 41.5% vs. 32.7% at week 96 (P = 0.076) and 43.8% vs. 32.8% at week 144 (P = 0.005). The difference reached statistical significance at the 144-week assessment (**Table 2**).

**Table 2:** Rates of successful immune reconstitution.

Characteristic	EFV-group	BIC/FTC/TAF-group	P
CD4 cell count ≥ 350 cells/μL, n (%)			
Week 48 (n=446)	76 (24.1%)	45 (34.4%)	0.027
Week 96 (n=439)	101 (32.7%)	54 (41.5%)	0.076
Week 144 (n=398)	99 (32.8%)	42 (43.8%)	0.050

### 3.3 Age-stratified analysis of CD4 cell count recovery

Although there was no statistically significant difference in baseline age between the two groups, the proportion of patients aged ≤50 years was numerically higher in the BIC/FTC/TAF group than in the EFV group (58.0% vs. 48.3%, P = 0.06). Given the potential influence of age on immune recovery, exploratory age-stratified analysis was performed.

#### 3.3.1 Subgroup aged ≤50 years

Switching baseline CD4 cell counts were comparable between the BIC/FTC/TAF and EFV groups (median [IQR]: 268.00 [223.50–299.25] vs. 275.50 [227.75–307.25]). At weeks 48, 96, and 144, median CD4 counts were significantly higher in the BIC/FTC/TAF group (respectively 335.00 [267.00–400.25] vs. 305.50 [244.75–362.25], P = 0.028; 359.00 [289.00–423.75] vs. 321.50 [256.50–396.25], P = 0.008; and 368.00 [306.00–441.00] vs. 332.00 [264.00–398.00], P = 0.006). The median increases from switching baseline were also significantly greater in the BIC/FTC/TAF group at all time points: 74.50 (34.50–129.50) vs. 37.50 (-7.25–84.50) at week 48 (P < 0.001), 100.50 (41.00–172.00) vs. 55.00 (4.50–107.00) at week 96 (P = 0.001), and 99.00 (47.50–184.50) vs. 59.00 (11.00–112.00) at week 144 (P < 0.001) (**Table 3**).

**Table 3:** Age-stratified comparison of CD4 cell count recovery between the EFV and BIC/FTC/TAF groups.

Age ≤ 50 years (n=228)		EFV-group (n=152)	BIC/FTC/TAF-group (n=76)	p
CD4 cell count	at switch	275.5(227.8, 307.3)	268.0 (223.5, 299.3)	0.405
	Week 48	305.5 (244.8, 362.3)	335.0 (267.0, 400.3)	0.025
	Week 96	321.5 (256.5, 396.3)	359.0 (289.0, 423.8)	0.008
	Week 144	332.0 (264.0, 398.0)	368.0 (306.0, 441.0)	0.006
Change in CD4 cell count	Week 48	37.5 (-7.3, 84.5)	74.5 (34.5, 129.5)	<0.001
	Week 96	55.0 (4.5, 107.0)	100.5 (41.0, 172.0)	0.001
	Week 144	59.0 (11.0, 112.0)	99.0 (47.5, 184.5)	0.001
Age>50 years (n=218)		EFV-group (n=163)	BIC/FTC/TAF-group (n=55)	p
CD4 cell count	at switch	258.0 (205.0, 300.0)	225.0 (193.0, 292.0)	0.064
	Week 48	272.0 (230.5, 335.0)	275.0 (211.5, 335.0)	0.980
	Week 96	291.0 (230.0, 360.0)	260.0 (213.5, 350.5)	0.330
	Week 144	286.0 (227.0, 354.0)	245.0 (202.0, 335.0)	0.455
change in CD4 cell count	Week 48	26.0 (-20.5, 65.5)	56.0 (7.5, 92.5)	0.049
	Week 96	40.0 (-5.0, 99.0)	61.5 (5.0, 103.3)	0.363
	Week 144	31.0 (-9.0, 94.0)	72.0 (-8.0, 101.0)	0.355

#### 3.3.2 Subgroup aged >50 years

In this subgroup, no statistically significant differences in median CD4 cell counts were observed between the BIC/FTC/TAF and EFV groups at weeks 48, 96, or 144. However, the median increase from switching baseline was significantly greater in the BIC/FTC/TAF group at week 48 (56.00 [7.50–92.50] vs. 26.00 [-20.50–65.50], P = 0.049). This difference was not consistently observed at later follow-up time points (**Table 3**).

### 3.4 Virological suppression outcome

In the EFV group, three patients had HIV-RNA >50 copies/mL at week 48. At week 96, one patient had HIV-RNA >50 copies/mL. By week 144, three patients had HIV-RNA >50 copies/mL, all of whom maintained levels <1000 copies/mL. The virological suppression rates were 99.4% (312/315) at week 48, 99.7% (308/309) at week 96, and 99.0% (299/302) at week 144. In the BIC/FTC/TAF group, no patients had HIV-RNA >50 copies/mL at either week 48 or 96, indicating virological suppression rates of 100%. At week 144, one patient had a detectable HIV-RNA level of 739 copies/mL, and the virological suppression rate was 99.0% (95/96).

## 4 Discussion

In this real-world retrospective cohort study in southwestern China, we evaluated the prevalence of INRs among PLWH receiving long-term antiretroviral therapy and explored longitudinal immune recovery patterns following switching from long-term EFV-based regimens to BIC/FTC/TAF in virologically suppressed INRs. Three main findings emerged. First, INRs remained highly prevalent, accounting for nearly one-third of long-term treated PLWH in Chongqing. Second, switching from long-term EFV-based regimens to BIC/FTC/TAF was associated with greater CD4 cell increases during follow-up compared with continued EFV-based therapy. Third, this association appeared more evident among patients aged ≤50 years.

Despite sustained virological suppression, incomplete immune reconstitution continues to pose a major clinical challenge. A cohort study reported an INR rate of 25.8% in Africa versus 10.8% in the United States (5). Other reported rates include 20.0% in South Africa (21), 24.7% in Ethiopia (22), and 40.8% in Guangzhou, China, respectively (23). In this study, we report an INR rate of 29.9% in Chongqing, China. This discrepancy likely reflects late presentation and initiation of treatment in Chongqing, which remains common in southwestern China. Given the established association between INR rate and increased long-term morbidity and mortality, further studies exploring management approaches for this population are warranted.

Several hypotheses may explain the observed longitudinal differences in CD4 recovery. First, among virologically suppressed PLWH, switching to an INSTI-based regimen may contribute to more stable long-term virological suppression and lower levels of residual viral replication, which have been associated with reduced immune activation and more favorable immune recovery in previous studies (19, 20, 24). In the present study, virological suppression remained high in both groups, while episodes of detectable viremia and low-level viremia were uncommon in the BIC/FTC/TAF group. Although this possibility was not specifically evaluated, differences in long-term virological control may have partially contributed to the observed changes in immune parameters. Second, the observed findings may also partly reflect discontinuation of long-term EFV exposure rather than a direct immunological effect of BIC itself. Previous studies have suggested that long-term exposure to older ART regimens may be associated with mitochondrial dysfunction, persistent immune activation, and impaired immune recovery (13, 14, 25–28). However, the biological mechanisms underlying the present findings remain uncertain, and causal interpretations should be avoided given the retrospective non-randomized design of this study.

Age significantly affected immune outcomes in this study. Among INRs aged ≤50 years, switching to BIC/FTC/TAF resulted in sustained and greater CD4 cell gains, whereas this advantage was attenuated in older patients. This observation can be explained by biological mechanisms. Firstly, the thymus serves as the primary lymphoid organ for the generation of T-cell subsets, especially naïve CD4 and CD8 T cells. While ART can enhance the thymic output of naïve CD4 cells and support immune reconstitution, thymic function declines with age due to progressive atrophy, leading to diminished production of new T cells (25, 26). Secondly, aging is associated with deterioration of the bone marrow microenvironment and stem cell senescence, which collectively impair the generation of functional immune cells (27). Furthermore, aging is linked to a state of chronic immune activation, which further disrupts effective immune reconstitution (28).

In recent years, the CD4/CD8 ratio has been regarded as a marker of immunosenescence and long-term immune recovery in PLWH (29, 30). Previous observational studies have suggested that INSTI-based regimens may be associated with more favorable CD4/CD8 ratio normalization compared with other ART classes (30–32). In the present study, although absolute CD4/CD8 ratios were not significantly different between groups throughout follow-up, greater increases from switching baseline were observed in the BIC/FTC/TAF group at weeks 96 and 144. These findings should be interpreted cautiously, and their clinical significance remains uncertain. Further studies incorporating detailed immune functional assessments are needed to better understand the potential relationship between ART regimen composition and CD4/CD8 recovery.

Current evidence does not support routine ART optimization solely to improve immune recovery among virologically suppressed PLWH. However, most available data have been generated in populations receiving contemporary ART regimens and may not fully reflect long-term EFV-treated populations. Given the widespread historical use of EFV-based regimens in China and the distinct clinical characteristics of long-term treated INRs, the present findings suggest that immune recovery patterns following optimization from long-term EFV-based regimens warrant further investigation. These observations should be considered exploratory and hypothesis-generating rather than evidence of a definitive therapeutic effect.

This study had several limitations. First, because of the retrospective observational design and non-randomized treatment switching, residual confounding and selection bias cannot be excluded. Second, loss to follow-up was primarily related to continuation of care at other institutions; however, attrition bias cannot be completely excluded. Third, multiple statistical comparisons across endpoints and time points increased the possibility of type I error, particularly for borderline P values. In addition, detailed immune functional assessments were unavailable. Therefore, the present findings should be interpreted cautiously and regarded as exploratory.

In this observational cohort of virologically suppressed INRs receiving long-term EFV-based therapy, switching to BIC/FTC/TAF was associated with greater longitudinal CD4 recovery, particularly among patients aged ≤50 years. However, given the retrospective non-randomized design, these findings should be interpreted cautiously and regarded as exploratory. Prospective studies are required to further clarify the potential relationship between ART regimen optimization and immune recovery in INRs.

## Data Availability

The original contributions presented in the study are included in the article/supplementary material. Further inquiries can be directed to the corresponding author.
